# De Profundis Clamavi—Out of the Sewers

**Published:** 1873-06

**Authors:** 


### Editorial.

### "De Profundis Clamavi"—"Out of the Sewers."

For several months past, popular patience, well nigh exhausted by the inefficiency and negligence of the Board of Health, has been gradually but certainly giving place to popular indignation, which, with the advance of the season, and consequent increased risk to public health, has been more and more decided in its demands for the abolition of this muncipal monstrosity, this sanitary abortion, miscalled a Board of Health. Stimulated by the lash of public opinion, the Board has at last essayed to do something—what? Honest sanitary work? No! Something to protect public health? No! Only to make a "report," to find an excuse for its own imbecility in the alleged inefficiency of another branch of the muncipal government. This is simply contemptible.

When the Board stands arraigned before the tribunal of public opinion for willful negligence, for which its only alternative plea is gross ignorance, it evades the issue altogether, and attempts to assign as the efficient cause of the great mortality of the city, insufficient sewerage.

And gravely attempts to demonstrate by figures that the man who has 14.50 feet of sewerage is safe, in comparison with him who has only 9.99 feet of this sanitary panacea. Upon such a theory as this, what a perfect temple of hygeia was the "Old Armory," where the proportion of sewer, *per capita*, was unlimited.

However, to give the devil his due, "the report" does not attribute the high rate of mortality *solely* to lack of sewerage, but assigns a portion of it to the "rain-fall." This is lucky for the Board of Public Works, and places a portion, at least, of the responsibility upon shoulders somewhat better able to bear it.

It would seem, then, from this "report," that the result of the operations of this Board of Health since 1867, carried on at an expense of half a million dollars more or less to the city, has been the discovery, that the cause of the great mortality of Chicago is insufficient drainage and too much rain. The JOURNAL moves that the "report" be accepted; that the Board of Public Works be requested to construct for each inhabitant his due proportion of 14.50 feet, or whatever it may be, of sewer; that the clergy be requested to pray for dry weather; and, above all things, that this Board of Health be discharged from further consideration of the subject. W. H.

